# Hepatitis E Antibodies in Laboratory Rabbits from 2 US Vendors

**DOI:** 10.3201/eid2004.131229

**Published:** 2014-04

**Authors:** Leslie Birke, Stephania A. Cormier, Dahui You, Rhett W. Stout, Christian Clement, Merlin Johnson, Hilary Thompson

**Affiliations:** Louisiana State University Health Sciences Center, New Orleans, Louisiana, USA (L. Birke, C. Clement, M. Johnson, H. Thompson);; University of Tennessee Health Science Center, Memphis, Tennessee, USA (S.A. Cornier, D. You);; Louisiana State University, Baton Rouge, Louisiana, USA (R.W. Stout)

**Keywords:** Hepatitis E virus, herpes simplex 1 virus, rabbits, HEV, viruses, zoonoses

## Abstract

We tested laboratory rabbits from 2 US vendors for antibodies against hepatitis E virus (HEV); Seroprevalences were 40% and 50%. Retrospective analysis of an ocular herpes simplex 1 experiment demonstrated that HEV seropositivity had no effect on experiment outcome. HEV probably is widespread in research rabbits, but effects on research remain unknown.

Hepatitis E virus (HEV) is a single-stranded RNA virus in the family *Hepeviridae* (*1*). HEV is transmitted through the fecal–oral and bloodborne routes and typically causes transient hepatitis; however, such infections can be fatal, especially in pregnant women (20% mortality rate) and in immunocompromised persons (*2*,*3*).

HEV has 4 genotypes, and genotypes 3 and 4 are found in humans and other animals (*1*). The genomic sequence of US rabbit HEV (GenBank accession no. JX565469) has been analyzed and the sequence places rabbit HEV as a distant member of the zoonotic HEV genotype 3 (*4*). A study in France found HEV in farmed and wild rabbits and characterized a closely related human strain of HEV (*5*). Although HEV has been detected in rabbits from Asian and European counties, researchers previously thought that HEV was not a major presence in rabbits in the United States (*5*–*7*). A recent study identified a 36% prevalence of HEV antibodies in animals on 2 rabbit farms in Virginia (*1*).

Because HEV may be a confounding factor in research and is a potential zoonotic pathogen (*5*), we tested rabbits from 2 US suppliers for HEV seroprevalence. Supplier A was a local conventional rabbit farm, and supplier B was a commercial vendor of specific pathogen free (SPF) research rabbits. Our hypothesis was 3-fold: 1)HEV is a possibly undiagnosed pathogen in laboratory rabbits, 2) HEV seroprevalence is unlikely in rabbits from supplier B (a SPF supplier), and 3) HEV seroprevalence might confound research. To investigate the latter hypothesis, we retrospectively analyzed records from a subset of animals used in a herpes simplex 1 virus (HSV-1) research project.

## The Study

### Animals

Two shipments of New Zealand White rabbits (70 rabbits shipment 1 [rabbit nos. AN1–AN20 and AH1–AH30] in June 2012 to Louisiana State University Health Sciences Center [LSUHSC]; shipment 2 [rabbit nos. AH31–AH51] in October 2012) were obtained from supplier A at LSUHSC. Supplier A also provides rabbits to the meat production industry. A third shipment comprising 10 Dutch Belted SPF rabbits (nos. DB1–DB10) were obtained from supplier B in August 2012 at LSUHSC. Supplier B rabbits were SPF for *Pasturella multocida*, *Pasturella pneumotropica*, *Bordetella brochiseptica*, *Treponema cuniculi*, *Clostridium piliformis*, cilia-associated respiratory bacillus*, Salmonella* spp*.*, *Encephalitozoon cuniculi*, *Eimeria stiedae*, *Eimeria magna*, *Eimeria intestinalis*, *Eimeria irresidua*, *Eimeria flavescens*, *Eimeria piriformis*, *Toxoplasma gondii*, *Passalurus ambiguous*, other helminths, parainfluenza virus 1, parainfluenza virus 2, reovirus, *Psoroptes cuniculi*, *Cheyletiella parasitovorax*, *Leporacarus gibbus*, and *Dermatomycosis* spp.

The rabbits’ care and use were approved by the LSUHSC Institutional Animal Care and Use Committee and were consistent with the Guide for the Care and Use of Laboratory Animals (*8*) and the Animal Welfare Act (http://www.aphis.usda.gov/animal_welfare/). The LSUHSC is fully accredited by American Association for the Assessment of Laboratory Animal Care, Int. The animals were singly housed in stainless steel rabbit cages (Suburban Surgical, Wheeling, IL, USA). On the day the rabbits arrived, blood was collected from each rabbit and stored for HEV testing. Rabbits from vendor A were treated for *P. cuniculi* and *L. gibbus* with selamectin (Pfizer New York, NY, USA). After arrival, the rabbits were acclimated to the facility for 7 days. After this period, they were used for research protocols approved by the Institutional Animal Care and Use Committee.

### Sample Collection

Approximately 0.5–1 mL of blood was collected through the rabbits’ middle auricular artery with a 25-gauge butterfly catheter. The blood was placed into serum separator tubes for centrifugation. Serum was then placed into microcentrifuge tubes and stored at −20°C until analysis. Laboratory personnel changed gloves between blood collection from each rabbit and between contact with each sample when transferring serum.

### HEV Infection and Outcome of Rabbit Model of Ocular HSV-1

The records of 30 animals from supplier A (AH1–AH30) were obtained retrospectively from an investigator who used the rabbits in an HSV-1 ocular keratitis study. Survival data and HEV antibody status were examined statistically to determine whether HEV status had any effect on the survival of the rabbits in the study. Briefly, 4 different strains of HSV-1 with different virulence levels were tested, including MCK D-gK, ICP0-delete, 17 syn + mutant, and McKrae. Rabbits were infected with these viruses, and keratitis score and survival rate were recorded.

### ELISA

ELISA was performed by using the protocol from Cossaboom with a few modifications (*1*). Plates (Fischer Scientific, Waltham, MA, USA) were coated with 6 µg HEV antigen (Clone# rHEV-ORF2; Feldan Bio Inc., Quebec, QC, Canada), incubated with 100 µL of rabbit serum diluted 10-fold in blocking buffer, and detected with horseradish peroxidase–conjugated secondary antibody (goat anti–rabbit IgG diluted 1:1000, Southern Biotech, Birmingham, AL, USA). The plates were read at 450 nm and 540 nm for correction on a SpectraMax M2 plate reader (Molecular Devices, Sunnyvale, CA, USA).

### Statistical Analysis

ELISAs for HEV were performed in duplicate on each serum sample, and the results were averaged. A 1-way analysis of variance was performed to find the optical densities that were not statistically different from those of the negative controls. The values of the negative controls and the negative samples were then used to determine the cutoff points for the ELISA (mean of the negative samples + 3 SDs).

To evaluate the possible effect of HEV serology status on the survival of rabbits used in the HSV-1 study, we used the Fisher exact test when HSV-1 survival status was cross-tabulated with the HEV serology status. Odds ratios are also reported (SAS, version 9.3, SAS Institute, Cary, NC, USA); α was set at 0.05.

### Prevalence of HEV in US Laboratory Rabbits

**Figure 1:**
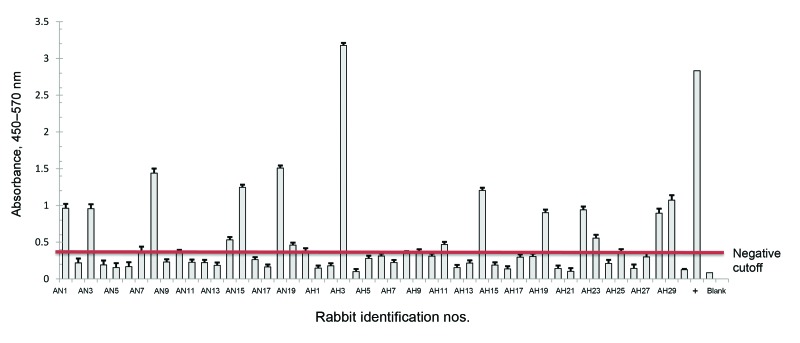
Hepatitis E virus antibody detection for rabbits from supplier A, shipment 1 (June 2012, Louisiana State University Health Sciences Center). Rabbits were purchased from supplier A. Serum was isolated and antibodies in the serum were measured by using ELISA. Rabbit identification numbers are listed on the *x*-axis and the mean optical density of each sample is listed on the *y*-axis. The negative cutoff point is indicated by the horizontal line.

**Figure 2:**
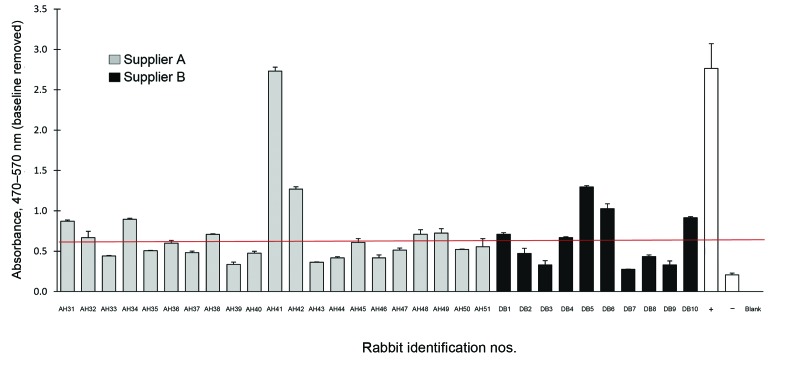
Hepatitis E virus antibody detection for rabbits from suppliers A and B, shipments 2 (October 2012, Louisiana State University Health Sciences Center [LSUHSC]) and 3 (8/2012 LSUHSC). Rabbits were purchased from supplier A (gray bars) and supplier B (black bars). Serum was isolated and antibodies in the serum were measured by using ELISA. Rabbit identification numbers are listed on the *x*-axis and the mean optical density of each sample is listed on the *y*-axis. The negative cutoff point is indicated by the horizontal line.

We found that the seroprevalence of HEV in the 70 rabbits from supplier A was 40%. The 10 rabbits from supplier B exhibited a 50% seroprevalence for HEV (Figures 1 and 2).

### HEV Infection and Disease Outcome of HSV-1–infected Animals

HSV-1 outcome results revealed no significant association between positive serologic HEV status and frequency of HSV-1 signs. Prevalence of positive HEV status was 32% (6/19) , compared with 36% (4/11) (p = 0.298, odds ratio 1.238, 95% CI 0.2592–5.9132) for frequency of HSV-1 signs.

## Conclusions

We sought to determine the seroprevalence of HEV in research rabbits within LSUHSC. We were surprised to identify HEV-seropositive rabbits from the SPF research rabbit supplier (supplier B) because of the strict health quality standards employed at this supplier. The difference in HEV seroprevalence between the 2 vendors may be a function of sample numbers. Testing a larger number of samples from supplier B would answer this question. HEV may be widespread in research facilities throughout the United States.

Statistically, we could not demonstrate any effect of HEV serology status on the survival outcome or keratitis scores of HSV-1–infected rabbits. However, the interpretation of these results is limited because only tested for HEV only by serologic testing and not reverse transcription PCR. Controlled studies in which rabbits are experimentally infected with HEV and then later infected with another pathogen are needed to determine the true effects of HEV co-infection on the outcome of systemic infection experiments in rabbits. 

Rabbits are commonly used in research and they often housed in a conventional setting. Because HEV is classified as a Biosafety Level-2 pathogen, wearing personnel protective protection equipment, such as masks, laboratory coats, and gloves, when working with rabbits is warranted (*9**,**10*). Laboratory animal care personnel, researchers, and support staff represent a new population at risk for HEV infection, and research facilities should be diligent in measures to prevention of this possibly zoonotic pathogen.
